# Kinship, favors, and officer integrity: exploring *wasta* in Kuwait’s National Police Force

**DOI:** 10.3389/fpsyg.2026.1849882

**Published:** 2026-08-17

**Authors:** Nasser AlSabah

**Affiliations:** 1 John Jay College of Criminal Justice, City University of New York Graduate Center, New York, NY, United States; 2 Kuwait National Police, Ministry of Interior, Kuwait, Kuwait

**Keywords:** Kuwait, police culture, police ethics, policing, *wasta*

## Abstract

**Introduction:**

*Wasta*, a culturally ingrained practice of favoritism prevalent in Arab culture, presents significant challenges to governance and organizational integrity in the Middle East. However, empirical research on its role in policing — an area notoriously tied to its use — is extremely scarce. This study proposes a framework explaining the elements that perpetuate *wasta* and examines officers’ attitudes toward the practice.

**Methods:**

A mixed-methods design was employed. Survey data were collected from 199 officers to identify correlates of attitudes toward *wasta*, and semi-structured interviews were conducted with a subsample of officers (*n* = 7) to explore the underlying dynamics shaping those attitudes.

**Results:**

Quantitatively, higher education and years of experience were significantly associated with officers’ critical views of *wasta* in police work. Qualitatively, catalysts of *wasta* involved bureaucratic inefficiencies and cultural expectations, while deterrents included personal barriers and organizational reforms such as digitization. Social pressures (e.g., familial and tribal expectations) emerged as the main drivers pushing individuals into participating in *wasta*.

**Discussion:**

The results underscore the need for comprehensive reforms — including the development of digital infrastructure, transparency initiatives, and ethics-focused training — to reduce *wasta* in policing and restore institutional integrity. Beyond Kuwait, this research contributes to broader discussions on informal governance practices and provides a basis for comparative studies in similar cultural contexts.

## 1 Introduction

*Wasta* arguably poses the most significant challenge for institutions in the Middle East when considered in relation to organizational justice and meritocratic values (AlAjmi et al., 2022; Al-Hiari, 2024; Alsarhan and Valax, 2020; Biaggi and Barkanian, 2020). Defined by Cunningham et al. (1994) as acts of mediation or intercession, *wasta* is a cultural construct deeply intertwined in Middle Eastern culture. Historically used as a means of settling differences among or within tribes, *wasta* served an integral diplomatic role, primarily functioning as a peacemaking instrument. Unfortunately, with the region’s modernization, the role of *wasta* in society has rapidly shifted from mediation to establishing advantages or priorities for certain individuals or groups across a wide spectrum of services or roles. This extends to regional politics (Doughan, 2017), critical public institutions (AlSabah, 2025a), and the private sector as well (Alsarhan and Valax, 2020). These literary works have overwhelmingly identified their role in deterring desirable organizational aspects, undermining organizational justice, and combating meritocratic values often associated with success and development. This is further exacerbated in the context of the criminal justice system, where equality and the rule of law serve as defining pillars. Although empirical literature has touched upon *wasta*’s involvement in policing in the Middle East and its association with police culture (AlSabah, 2025a,b), there remains a significant gap in theoretical frameworks explaining how or why it persists. This study contributes to addressing this gap by developing a conceptual framework—derived deductively from established theory and refined inductively through analytical analysis—that enables effective identification and future exploration of aspects associated with wasta, providing necessary information useful for understanding and deterring it.

### 1.1 *Wasta* and organizational justice

As prefaced in this article’s introduction, *wasta* serves as a boon to organizations, greatly symbolizing itself as an inhibitor of organizational justice and meritocracy (Al-Twal et al., 2024; AlSabah et al., 2026). This is especially true for countries with lengthy bureaucratic processes, making potential resolutions even more lucrative and desirable (Karolak, 2016). The contemporary introduction to the world of *wasta* begins in the pre-employment phase, significantly influencing the employment prospects of newly graduated individuals (Baranik et al., 2023). Afterward, its profound implications for organizational justice are evident, leading to low morale, reduced job satisfaction, and higher turnover rates (Alsarhan and Valax, 2020). Al-Twal et al.’ (2024) interviews with employees from various industries revealed that *wasta* has significant implications for distributive, procedural, and interactional justice in organizations, fostering a sense of hopelessness for individuals unable to transcend its influence. This results from certain employees receiving preferential treatment in promotions and salary increases, even when lacking the necessary skills or performance outcomes (Ali et al., 2024).

These occurrences are further exacerbated when they are prevalent in critical governance institutions, eroding public trust in government, which is predicated on providing equal treatment (Jones, 2024). Alreshoodi (2018) examined the impact of *wasta* on the public sector in Saudi Arabia. Emphasizing the concept of public service motivation (PSM), he sought to explore whether the use of *wasta* was sufficient to mitigate the manifestation of such motivation. He found that *wasta* served a significant moderating effect on motivation across various areas (e.g., organizational commitment, job satisfaction), implying its negative role in the effectiveness and efficiency of government sectors. *Wasta*’s grasp also extends to public education, undermining the fairness of education systems in the Middle East. AlAjmi et al.’s (2022) study in Kuwait explored the interference of external power holders in the Kuwaiti education system. Semi-structured interviews with educational leaders revealed that *wasta* is prevalent among the pressures faced in education, undermining important educational outcomes and affecting generations of graduates. *Wasta* also extends to the region’s political processes, with efforts at democratization facing significant challenges. This is central to Doughan’s (2017) commentary on the issue, which examines the influence of wasta in Jordanian politics. Contrary to the expectation that democratization deters the perpetuation of wasta, Doughan argues that parliamentary politics in Jordan operates largely through it: constituents cast votes in anticipation of intercession, and elected officials, acting as conduits of wasta, press government bureaucrats to deliver services to their constituents in return.

Beyond the Arab world, similar concepts of *wasta* can be found in East Asia, where it is known as *guanxi*, described as networks of personal connections and relationships used for mutual benefit, involving trust, reciprocity, and the circulation of favors (see Hutchings and Weir, 2006). Like *wasta*, *guanxi* has been heavily criticized as a deterrent to meritocracy. Braendle et al. (2005) elaborated on the *guanxi* and its association with corporate governance in China. It highlights that while serving as a crucial element for building relationships and securing business successes, guanxi generally acts as a double-edged sword. This stems from its perpetuation of the exchange of favors, which strengthens informal networks that hamper formal governance structures and economic growth. Generally applicable, nepotism has been extensively studied across various fields, with Arasli and Tumer (2008) identifying nepotism, cronyism, favoritism, and unprofessional practices—such as giving preferential treatment to select individuals’ family and friends. Using survey data from 576 respondents, the findings indicate a positive relationship between workplace stress and dissatisfaction within organizations, with nepotism serving as the most stress-inducing.

While some argue that *wasta* is a pragmatic response to navigating bureaucratic inefficiencies and corruption prevalent in many Arab countries (Al-Ramahi, 2008), scholars predominantly view it as an obstacle to development and modernization, symbolizing stagnation tied to tribalism and outdated social structures (AlSabah et al., 2025b; Doughan, 2024).

### 1.2 Police integrity, culture, and informal institutions

Police corruption is highly detrimental to police institutions and their societal roles (AlSabah et al., 2026; Caldero et al., 2018; Punch, 2009). It influences judicial processes, recruitment practices, and the distribution of resources and personnel, compromising the fairness, impartiality, and efficiency essential to effective policing. This is particularly relevant within heavily bureaucratic systems, as found in the Middle East, where concentrated power dynamics can pose extreme impediments to effective governance, institutional integrity, and mechanisms for change when dictated by immoral actors (Ali and Weir, 2020). Such practices erode meritocracy and foster perceptions of injustice and corruption, significantly undermining public trust in law enforcement institutions and diminishing overall police efficacy.

Police integrity, crucial to effective policing, relies heavily on public trust in law enforcement (Hinds, 2009; Peyton et al., 2019; Worden and McLean, 2017). Bradford and Myhill's (2014) research in England and Wales found that public trust and confidence in the police were influenced by both instrumental and expressive factors. Their findings suggest that trust in the police is not solely based on their effectiveness or crime-reduction numbers but is heavily tied to social perceptions of order and integrity. Similarly, Miller and Davis (2008) concluded that perceptions of law enforcement were heavily influenced by attitudes toward police misconduct, which is highly relevant to the topic of *wasta*, as members of the public have come to stereotype officers as individuals peddling favors in the form of *wasta*. This has similarly been studied in Russian policing, with the label ‘predatory policing’ as an ingrained aspect of law enforcement culture, characterized by officers primarily serving their own interests rather than those of the public (Gerber and Mendelson, 2008).^1^ This is highly problematic, as the literature has often concluded that negative views of the police further perpetuate alienation between the public and law enforcement (Nix et al., 2020; Wolfe et al., 2024). Consequently, the pervasive nature of *wasta* not only compromises policing effectiveness but also jeopardizes the fundamental legitimacy and ethical standing of police institutions within Kuwaiti society.

Additionally, *wasta* can negatively impact police institutions, with research indicating the role of organizational justice in officer satisfaction and effectiveness (Gutschmidt and Vera, 2022; Myhill and Bradford, 2013). Debbaut and De Kimpe (2023) found that police legitimacy within institutions is closely linked to officers’ identification with professional standards and ethical leadership, a finding corroborated by Cockcroft (2014) in his research on police leadership and culture. However, the prevalent practice of *wasta* in Kuwaiti police agencies directly undermines these professional values, linking officers’ integrity to their participation in or resistance to it. Similar to traditional policing constructs such as masculinity and attitudes of ‘us vs. them’ (Brough et al., 2016), participation in *wasta* culture is often expected from newly sworn-in officers. Refusal to do so often introduces organizational pressures that persist for years, forcing unwilling participants to adhere to unwritten rules that inhibit proactive organizational change (Charman and Corcoran, 2015).

International research exploring constructs analogous to *wasta*—such as nepotism—provides further evidence of these negative impacts on police institutions. Similarly, Mutlu (2000) identified unethical connections between Turkey’s political elite and law enforcement, emphasizing nepotism and favoritism as significant obstacles to effective police organization. The study suggests that such relationships undermine democratic processes and contribute to the growth of organized crime groups. Xu et al. (2018) provide a framework through which *guanxi*, an East Asian equivalent of *wasta*, perpetuates corruption in China, identifying key psychological processes—including the normalization of corruption—that help conceal corrupt intentions. Key concepts discussed include establishing connections with bribees (e.g., through gift-giving) and mechanisms that normalize and justify corrupt acts, thereby reducing accountability and responsibility. Their findings are corroborated by the literature linking *guanxi* to law enforcement practices, which argues that it fosters inconsistent law enforcement outcomes and encourages organized criminal networks (Shi, 2024).

In Kuwait, *wasta’s* influence is profound, permeating administrative, judicial, and political spheres. Influential individuals utilize tribal and ethnic affiliations to gain or provide favors through employment, governmental approvals, and institutional promotions. Such systemic favoritism weakens public institutions, exacerbates corruption, and creates an environment in which police officers and judicial officials are hesitant or unable to enforce the law on individuals protected by *wasta*, given the potential personal and professional repercussions (see AlSabah et al., 2026). This is similarly true for government agents who impede the administration of justice, all to obtain or fulfill obligations associated with *wasta*. Furthermore, its prevalence within Kuwait’s police force has established an informal institution of favor-sharing, cementing a clientele system that poses a serious threat to the integrity of Kuwait’s police institutions and its criminal justice system.

### 1.3 Current study

This study aims to adopt a mixed-methods approach to establish a conceptual understanding of *wasta*, exploring potential correlates of attitudes toward *wasta* quantitatively while qualitatively collecting nuanced insights into the elements that perpetuate its use within Kuwait’s police institutions. Drawing on Bourdieu’s (1977, 1986; Bourdieu and Wacquant, 1992) *Theory of Practice*, this study proposes a conceptual framework centered on three interrelated components: circumstances, capacity, and pressures. These components explain both the initiation and provision of *wasta*, underscoring mechanisms central to its perpetuation and forming pillars against which potential findings are discussed in conjunction.

The findings can not only strengthen and reinforce the proposed framework but also inform policies to reduce wasta in institutions that require impartiality and meritocracy. By pinpointing structural gaps and cultural drivers, the study offers actionable reform recommendations. It also adds to broader discussions on corruption, governance, and norms in the Middle East, serving as a basis for comparative studies on informal practices elsewhere.

## 2 Methods

This study employs a mixed-methods approach that combines survey analysis with semi-structured interviews to elaborate on and offer insights into concepts considered crucial not only to the perpetuation of *wasta* but also to the factors that provoke its use. However, before starting data collection, it is essential to take the necessary steps to create a pathway for accurately assessing and elaborating on these concepts. This begins with analyzing previous frameworks that can help explain this phenomenon, one that encapsulates its core concepts.

### 2.1 Theoretical framework

To achieve the core research goal of this study, the methodology used in this article draws on validated, established frameworks that address similar issues related to *wasta*.

Pierre Bourdieu’s theory of practice (Bourdieu’s, 1977, 1986; Bourdieu and Wacquant, 1992) provides a sociological framework for understanding how informal behaviors like *wasta* are rooted in social structures, internalized dispositions, and access to various forms of capital, such as police authority. His core concepts—field, capital, and habitus—serve as key analytical tools. In the wasta context, the ‘field’ (Bourdieu and Wacquant, 1992) is the institutional setting, such as police organizations, where officers compete for influence, advancement, or resources.

‘Capital’—especially social and symbolic capital (Bourdieu, 1986)—includes personal networks, status, or affiliations used to gain informal advantages. Bourdieu (1986) defines social capital as resources from enduring networks of mutual acknowledgment, which closely relates to *wasta*’s reliance on established relationships. Successful intercession both utilizes and reinforces these networks, creating a cycle of obligations.

‘Habitus’ (Bourdieu, 1977) refers to internalized norms and expectations that shape a person’s sense of obligation, loyalty, and willingness to engage in practices such as *wasta*. This concept is linked to *wasta* because its use often aims to benefit one’s kin or tribal group, with individuals risking social exclusion if they do not provide such favors. These concepts explain how *wasta* is practiced and why it becomes normalized and sustained within organizational cultures over time.

### 2.2 Conceptual *wasta* framework

**Figure 1:**
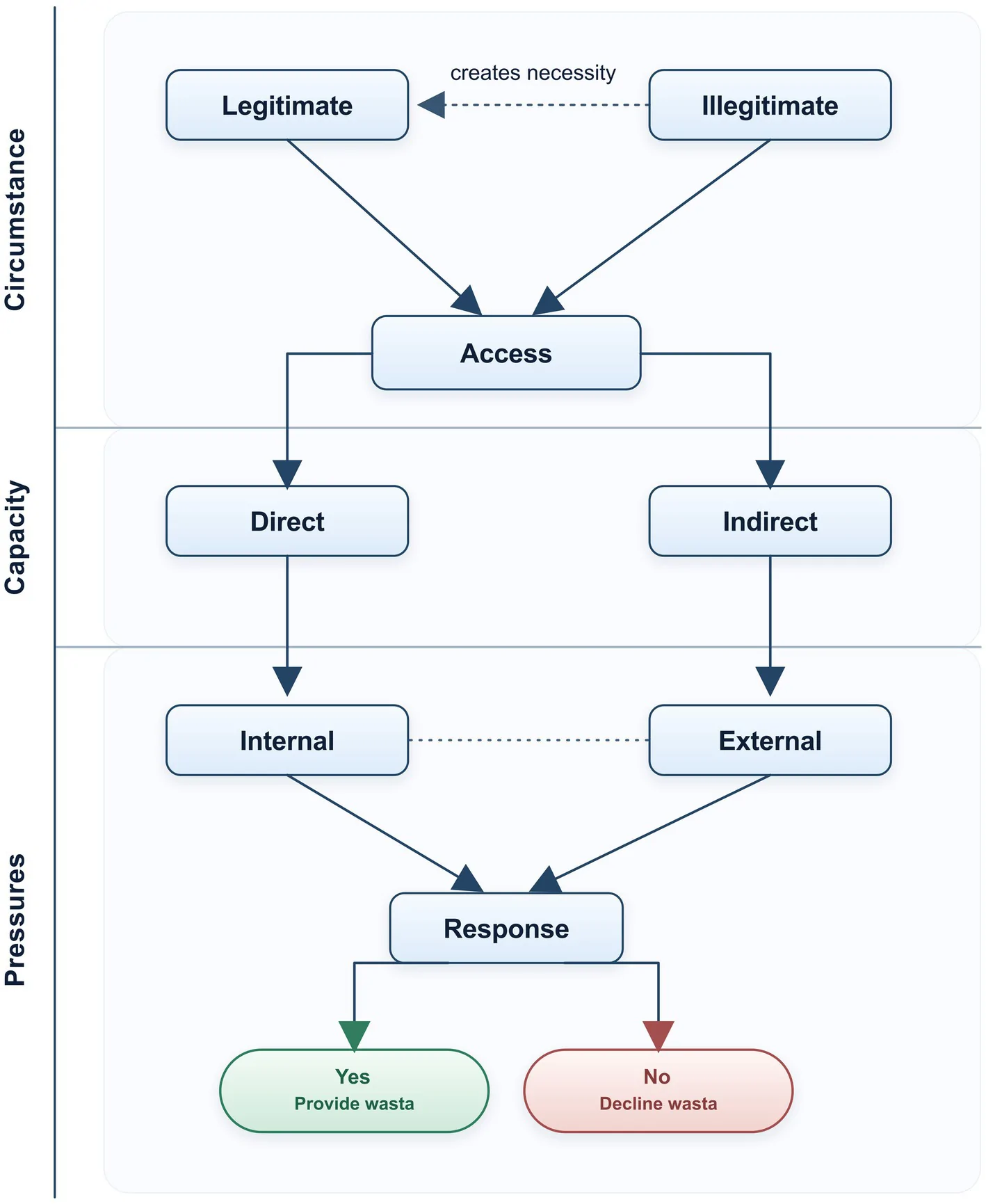
Framework for *wasta*. The following figure identifies the core concepts associated to *wasta*, a framework that hypothesizes the core concepts behind the perpetuation and reasoning behind the use of *wasta*. For the first section, two core concepts exist: circumstances and capacity. For the latter section, one core concept concerns providers’ role in *wasta*, mainly highlighted by the pressures and underlying concepts attached to it.

The theory enables the construction of a potential conceptual model—*wasta* as a strategic practice within a socially and institutionally structured field. Serving as platforms for this current framework, mechanisms central to its perpetuation serve as pillars. This framework (detailed in Figure 1) was deductively developed from Bourdieu’s (1977, 1986; Bourdieu and Wacquant, 1992) theory. The proposed framework comprises three interrelated components—circumstances, capacity, and pressures.

(1) Circumstances: To provide a comprehensive understanding of *wasta*, it is necessary to understand why individuals resort to it. Cunningham et al. (1994) provide an accurate representation of two forms of *wasta* most used in current-day contexts: ‘intercessory’ or ‘intermediary’ *wasta* (Cunningham et al., 1994). These two instances account for contemporary reasoning behind the initiation of *wasta*, conceptualized as (1) illegitimate and (2) legitimate in the context of this study. Shortcomings in institutions or a lack of belief in them serve as catalysts for individuals to seek the use of *wasta*, often reinforcing an ecosystem in which *wasta* is required rather than optional (Al-Twal and Aladwan, 2020). Descriptions and examples for both will be provided for each:

- Illegitimate: identified as instances where *wasta* is used unjustly. When individuals can achieve the desired outcomes or results but regard the use of *wasta* as a means of doing so more quickly (e.g., finishing paperwork that often takes a month in a few days; skipping the line). This can also extend to acquiring treatment that is otherwise unwarranted (e.g., avoiding a speed ticket).
- Legitimate: Although unofficial, *wasta* is often considered a last resort for individuals when all formal means of securing what is warranted are blocked or rebuffed (e.g., paperwork is deliberately delayed). *Wasta* brokers often use these instances to set the stage for their intervention, collecting favors in the process. It should also be noted, as identified by the dotted line in Figure 1, that illegitimate use of *wasta* often serves as the most common driver of legitimate means of *wasta* (see Elsaher, 2024).

(2) Capacity: Circumstances discussed previously have led to attempts to contact someone who can provide the service of *wasta*. This can be a family member, a tribal member, or even an acquaintance. These individuals can be either someone who directly provides outcomes requested by the client (e.g., the person writing the citation) or someone who has access to that individual (e.g., the partner of the individual writing the citation). The closer an individual is to the outcome, the better their chances of successfully completing a *wasta* transaction.

(3) Pressures: Like aspects forcing individuals to use *wasta*, pressures similarly exist upon those requests that are directed at. It is only after accounting for these influences that a person decides whether to provide *wasta*, ultimately determining their response to those who request it. These pressures can come in two forms:

- Internal pressures arise when internal influences mount pressure on individuals capable of providing *wasta*, whether for good intentions (e.g., assisting individuals who have been negatively affected by others’ use of *wasta*) or nefarious intentions (e.g., collecting favors to advance personal ambition).
- External pressures, instances where participation in *wasta* is influenced by external forces (e.g., tribal or ethnic obligations prevalent in Arab society). These two influences are not mutually exclusive and are often evident in decisions.

Although the three pillars are presented as separate components, they are conceptually interconnected through a sequential demand-and-supply logic: circumstances influence the demand for *wasta* by shaping whether informal intercession seems expedient or necessary; capacity affects whether that demand can realistically be met, based on the seeker’s proximity to capable actors; and pressures influence the provider’s decision, converting cultural and organizational obligations into the final supply of *wasta*.

### 2.3 Quantitative data collection

#### 2.3.1 Data

The survey data for this study were drawn from two datasets on police personnel at Kuwait’s police stations. Although collected separately, both surveys included similar sections on *wasta* in policing and officers’ opinions of the public. Additionally, demographic, professional, and geographical data were collected in both surveys. These similarities provided a basis for merging the datasets, enabling a comprehensive analysis of constructs consistently measured in both sources. This culminated in a sample consisting of survey responses from 205 on-duty officers in Kuwait, the largest to explicitly examine *wasta* in a criminal justice system in the Middle East.

The first dataset originated from a corresponding study conducted by AlSabah et al. (2025a), which assessed leadership traits among police leaders and their associations with various policing themes using 48 items. This dataset included 60 observations from senior police station leaders across Kuwait’s 72 police stations (83% response rate). Non-probability total population sampling was used for this dataset due to the small size of the leadership population in Kuwait’s police stations. It should be noted that this study uses this data to address research questions previously unexplored in the original study, which simply assessed whether attitudes among leaders correlated with leadership types.

The second dataset came from the KNP’s Public Security sector and sampled non-leadership officers (*n* = 145). It used a 19-item survey comprising six sections to assess attitudes toward policing. Two police stations from five provinces in Kuwait were designated using stratified cluster sampling, resulting in 10 stations having their officers randomly sampled. Stations in Kuwait deploy approximately 35–50 officers each, serving as continuously operating hubs for security and crime deterrence across Kuwait’s neighborhoods and areas.

It is worth noting that police participants were all male. Female officers are not currently stationed at police stations in Kuwait, where the survey collection was done. Limitations related to this are explored in this study’s limitations section.

#### 2.3.2 Analytical strategy

This study preliminarily conducts multivariate testing to ascertain potential relationships between demographics and our primary outcome variable, officers’ attitudes toward *wasta* and its negative influence on policing. Three main steps were taken to establish this as a successful analytical strategy.

Our dataset, merging two sources, required cleaning. Officers’ ranks varied widely, so we created a four-category rank variable: low/non-commanding (NCO/1), high/non-commanding (NCO/2), low/commanding (CO/1), and high/commanding (CO/2). These distinguished non-commanding officers were trained for at least 6 months under commanding officers with 4 + years at Kuwait’s police academy. The two datasets—one sampling first-line officers, the other station leaders and middle-tier officers—enabled us to add a binary leadership variable (1 = leader). The experience variable, based on broad estimates, was restructured into ordinal categories (0–5, 5–10, 10+ years). Finally, data from Mubarek AlKabeer province (*n* = 6) were removed to improve model reliability. Our final sample consisted of 199 survey responses, with an average age of 35 (SD = 8.24), ranging from 19 to 57 years. 35% of the sample had college degrees, while 27.5% held leadership positions in their respective stations. Officer ranks were distributed as follows: low non-commanding officers (21.6%), high non-commanding officers (12.6%), low commanding officers (14.6%), and high commanding officers (51.3%). Most of the officers sampled (65%) had more than 10 years of service, 20% had between 5 and 10 years, and 15% had fewer than 5 years. Before factor analysis, perception scores indicated moderate levels for *Wasta* Culture (*M* = 12.48, SD = 4.30) and Public Sentiment (*M* = 18.18, SD = 4.48). The largest number of officers worked in Kuwait’s capital, Asma (38%), followed by Jahra (20.5%), Hawalli (19.5%), Ahmadi (12%), and Farwaniya (10%). Full summary statistics are provided in Table 1.

**Table 1:** Descriptive statistics.

Variable	Obs	Mean	Std. dev.	Min	Max
Age	199	35.03	8.238	19	57
Education (1 = college)	199	0.35	0.478	0	1
Leadership (1 = head of unit)	199	0.275	0.448	0	1
Rank					
Low Non-commanding Officers	43	0.216	0.413	0	1
High Non-commanding Officers	25	0.126	0.332	0	1
Low Commanding Officers	29	0.146	0.354	0	1
High Commanding Officers	101	0.513	0.501	0	1
Experience					
0–5 years	30	0.15	0.358	0	1
5–10 years	40	0.20	0.401	0	1
10 + years	129	0.65	0.478	0	1
Perceptions					
*Wasta* Culture	199	12.48	4.297	5	24
Public Sentiment	199	18.18	4.479	6	29
Area of Work					
Ahmadi	24	0.12	0.326	0	1
Asma	75	0.38	0.487	0	1
Farwaniya	20	0.1	0.301	0	1
Hawalli	39	0.195	0.397	0	1
Jahra	41	0.205	0.405	0	1

Participating officers’ demographical, professional, perceptional, and geographical data.

Secondly, structural identification techniques were required to establish valid constructs to address this study’s research goals. This pertained to the 12 survey items included in the dataset, comprising two sections of six items each, representing wasta and public perceptions. Survey items aimed to assess officers’ views on these topics, gauging whether they held favorable or unfavorable opinions. It should be emphasized that these items captured officers’ attitudes toward wasta rather than their self-reported participation in the practice; all subsequent interpretations are framed accordingly.

Exploratory factor analysis (EFA) was conducted to derive composite variables that capture the latent constructs underlying these categories. Because all items were measured on five-point Likert scales, polychoric rather than Pearson correlations were used, and factors were extracted using maximum likelihood with oblique (oblimin) rotation, the latter selected because the two constructs were theoretically permitted to correlate. The correlation matrix was suitable for factor analysis [KMO = 0.73; Bartlett’s test of sphericity, *χ*^2^(66) = 840.21, *p* < 0.001]. Items were retained where their primary loading reached 0.40, applied iteratively such that the weakest sub-threshold item was removed and the solution re-estimated before any further exclusion.

Two items were removed under this criterion. One item from the wasta section was excluded for an unacceptable loading (0.018), while a second item was subsequently removed from the perceptions section (0.334). It should also be noted that a single item, “Wasta negatively affects police work,” was a borderline exemption to the threshold mentioned above due to the borderline nature of its loading value and its direct association with the topic being investigated. The retained ten-item solution yielded two factors, both satisfying the Kaiser criterion: perceptions of the public (*λ* = 2.632) and wasta (λ = 2.202), together accounting for 48.3% of item variance.

The two constructs were empirically distinct (factor correlation *r* = 0.109), indicating valid discriminant validity. Composite reliability was satisfactory for both factors (CR = 0.782 and 0.835). Internal consistency was acceptable for perceptions of the public (*α* = 0.799) and marginally below convention for wasta (*α* = 0.695). Average variance extracted met the conventional 0.50 threshold for perceptions of the public (AVE = 0.518) but not for wasta (AVE = 0.434). Although lacking in some goodness-of-fit measures, the novel nature of the study and topic in hand—one that has been significantly scarce in criminal justice—coupled with the borderline nature of these measures, provided reason to retain them. Table 2 presents the categories, the items under each category, each item’s factor loading, and the finalized eigenvalues.

**Table 2:** Survey item factor loadings.

Variable	Survey item	Factor loading	Eigen value
Wasta	(1) Wasta negatively affects police work.	0.391	2.202
	(2) Members of the police exploit wasta negatively.	0.840	
	(3) Members of the police place personal connections over the enforcement of the law.	0.812	
	(4) The public often needs to use wasta to have justice.	0.585	
	(5) Members of the police exploit their authority for personal gain.	0.556	
Perceptions of the Public	(1) I often believe the intentions of members of the public are good.	0.417	2.632
	(2) Members of the public respect the law.	0.605	
	(3) The public treat the police with respect.	0.825	
	(4) Interactions with the public are often polite.	0.906	
	(5) Members of the public are cooperative with the police.	0.741	

The following table lists survey items used for this study (*n* = 10) and the topic areas under which they are categorized. Also included are factor loading values culminating in two distinct factor variables, (1) *Wasta* and (2) Perception of Public and their corresponding Eigenvalues. A threshold of >0.4 was applied to factor loadings for exclusion.

Cumulative variance 48.3%. Factor correlation *r* = 0.109, RMSEA = 0.097, TLI = 0.882. Wasta: *α* = 0.695, AVE = 0.434, CR = 0.782. Perceptions of the Public: *α* = 0.799, AVE = 0.518, CR = 0.835.

Lastly, necessary diagnostics were conducted to maximize the validity of linear models. This included tests of heteroskedasticity, multicollinearity, among other assumptions of linear regression. Upon conducting the necessary pre-diagnostic checks for normality, the models’ residuals consistently violated normality. Hence, Ordinary Least Square (OLS) models with robust standard errors (SE) were used in the final analysis. Influential outliers were removed via Cook’s *D*, with observations exceeding the threshold of 4/n considered influential and subsequently excluded from the final analysis. The final sample sizes used are listed in their respective models (see Table 3).

**Table 3:** Interview participants.

Interviewee	Age	Rank	Years of experience	Sector	Interview length
Officer 1	38	Lieutenant Colonel	17	Public Security (administration)	21:20
Officer 2	37	Colonel	15	Investigations	17:10
Officer 3	30	Captain	8	Traffic	15:25
Officer 4	36	Colonel	12	Human Resources	16:35
Officer 5	35	Colonel	10	Public Security (precincts)	18:34
Officer 6	31	Captain	9	Border Patrol	17:49
Officer 7	29	Captain	7	Immigration	16:47

The following table lists officers that participated in interviews discussing *wasta* in the police force. This includes: interviewees and their ages, rank, years of experience, and sectors of work. Also included is the length of each interview.

All statistical analyses and table creation were conducted using STATA (Release 17; StataCorp LLC). The significance threshold was set at *p* < 0.05.

### 2.4 Qualitative data collection

The study’s qualitative component included semi-structured interviews with officers working in Kuwait’s police force. To ensure the quality and trustworthiness of the qualitative analysis, interviews were conducted until thematic saturation was achieved (Guest et al., 2006). This approach ensured that data collection was driven by depth rather than a predetermined number of participants. Once it became evident that further interviews yielded minimal new ideas or variations concerning *wasta*, data collection ceased.

To capture interview narratives, thematic analysis was used following Braun and Clarke’s (2006) six-phase framework: (1) familiarization, (2) coding, (3) searching for themes, (4) reviewing, (5) defining, and (6) reporting. Sub-themes emerged through iterative coding in NVivo, initially against parent themes (Capacity, Circumstances, Pressures). Recurring concepts and patterns were examined, expanding nodes into sub-themes when multiple references highlighted distinct aspects. For example, cultural norms within Pressures led to Societal Expectations, and ethical discussions led to Boundaries. This combined deductive (theory-driven) and inductive (data-driven) coding, ensuring alignment with theory and responsiveness to participants’ experiences. Because coding was done by a single researcher, several measures were implemented to ensure consistency: a structured codebook aligned with the conceptual framework was maintained throughout; all transcripts were recoded during a second full review to verify the stability of code application; and analytic memos documented coding decisions and emerging interpretations.

#### 2.4.1 Interview process

Interviews were conducted via private phone calls. Each session lasted approximately 20 min. Interviews were fully audio-recorded, allowing for a robust thematic analysis afterward. Transcripts or summaries were made available to participants upon request. The interview guide followed a thematic structure aligned with the study’s core conceptual pillars. Interview Questions are provided as Supplementary List 1.

In attempts to provide more comprehensive narratives regarding *wasta*, officers with more than 5 years of experience were targeted for inclusion in the criteria. Convenience sampling was employed to recruit willing participants, with efforts made to select officers from various areas of Kuwait’s police force. These sectors included both administrative (e.g., immigration and human resources) and field-centered (e.g., airport security, public security, investigations, etc.) areas of the police force. A total of 19 officers were preliminarily contacted for participation. Nine agreed to be interviewed; however, 2 declined to be audio recorded, hence their exclusion from the final sample. The final interview sample included 7 officers (37% participation rate), from 6 different sectors, were sampled before thematic saturation occurred. The average interview length was 17:40 min.

Saturation was monitored continuously by noting the appearance of new codes and sub-themes after each interview. It was considered achieved when the last two interviews revealed no new sub-themes and only reiterated existing codes. This aligns with research indicating that core themes tend to stabilize after a few interviews (Guest et al., 2006).

Interviews were conducted entirely in Kuwait’s native language, Arabic. Interview recordings were transcribed using Sonix, a transcription service that proved to be consistent and accurate for Arabic dialog. After transcription, Sonix’s translation capabilities were used to translate Arabic transcriptions into English text. Lastly, text was imported into Nvivo, a qualitative analysis software to highlight themes and codes in the text. After the interview, participants were thanked for their time and reminded that they could withdraw from participation or request the removal of their data at any point. They were also given the opportunity to pose any final questions or share additional thoughts. A summary of the interviewees and their descriptions is provided in Table 3.

## 3 Results

### 3.1 Survey data

Table 4 presents three nested models, organized by predictor block and their sequential integration according to data orientation. The predictors were incorporated in the following order: (1) demographic data (age and educational background) and professional background (leadership status, rank [baseline = NCO/1], and experience [baseline = 0–5 years]); (2) perceptions of the public; and (3) geographical data (area of work, baseline = Asma). This systematic approach enabled a comprehensive understanding of how various factors collectively relate to officers’ attitudes toward *wasta* in the policing context.

Model 1 reached conventional significance overall (robust Wald *F* = 2.12, *p* = 0.036) but explained only a modest share of variance (*R*^2^ = 0.068; adjusted *R*^2^ = 0.026), and individual estimates should be read with corresponding caution. Among individual predictors, officers with 5–10 years of experience were significantly more likely than newly sworn officers (0–5 years) to hold critical views of *wasta* (*β* = 0.593, *p* < 0.05). Officers with more than 10 years of experience showed a positive association of comparable magnitude, though this fell short of conventional significance (*β* = 0.481, *p* < 0.10). Neither age, educational background, leadership status, nor rank was significantly associated with the outcome at this stage.

**Table 4:** Nested regression models.

Predictor	Model 1	Model 2	Model 3
	*β (SE)*	*β (SE)*	*β (SE)*
Demographics			
Age	0.001 (0.012)	0.004 (0.011)	−0.001 (0.011)
Education	0.226 (0.144)	0.291* (0.143)	0.288* (0.142)
Professional background			
Leadership	−0.097 (0.198)	−0.146 (0.194)	−0.187 (0.220)
Rank			
NCO/2	−0.169 (0.241)	−0.044 (0.233)	0.054 (0.236)
CO/1	−0.217 (0.226)	−0.234 (0.223)	−0.241 (0.220)
CO/2	−0.044 (0.195)	−0.073 (0.190)	−0.001 (0.212)
Experience			
5–10 years	0.593* (0.231)	0.581* (0.228)	0.601** (0.226)
10+ years	0.481^†^ (0.255)	0.427^†^ (0.246)	0.610* (0.251)
Perceptions			
Public Sentiment	–	0.163* (0.069)	0.118^†^ (0.070)
Geographical			
Ahmadi	–	–	0.170 (0.211)
Farwaniya	–	–	−0.076 (0.240)
Hawalli	–	–	0.341^†^ (0.189)
Jahra	–	–	0.037 (0.199)
Intercept	−0.521 (0.342)	−0.610^†^ (0.336)	−0.663^†^ (0.339)
*R* ^2^	0.068	0.089	0.113
Adjusted *R*^2^	0.026	0.044	0.047
Robust Wald *F*	2.12*	2.56**	1.91*
*p* (*F*)	0.036	0.009	0.032
*N*	189	190	189

The following is a table exhibiting nested OLS regression models examining predictors of negative attitudes on *wasta* culture. The first level uses standard demographical information in age (in years) and education (1 = college), as well as officers’ professional background with leadership status (1 = assigned leadership position), rank (NCO/1, the lowest ranks serving as baseline) and experience (least experienced officers, 0–5 years, serving as baseline). Thirdly, perceptions in the form of officers’ attitudes of the public. Lastly, the Officers’ province of work of officers is introduced to the model, with Asma (Kuwait’s capital) serving as the baseline.

Introducing perceptions of the public improved model fit (Δ*F* = 4.13, *p* = 0.044), and this specification produced the strongest omnibus test of the three (robust Wald *F* = 2.56, *p* = 0.009; *R*^2^ = 0.089; adjusted *R*^2^ = 0.044). Educational background (1 = degree holders) became positively associated with critical views of *wasta* (*β* = 0.291, *p* < 0.05), whereas it had been non-significant in the preceding model. Officers with 5–10 years of experience remained significant (*β* = 0.581, *p* < 0.05), while the 10+ years category again approached but did not reach conventional significance (*β* = 0.427, *p* < 0.10). Officers’ perceptions of the public were positively associated with the acknowledgment of *wasta* as a detrimental construct in policing (*β* = 0.163, *p* < 0.05).

Integrating geographical data did not introduce any new significant predictors, and the geographical block did not significantly improve model fit (Δ*F* = 1.09, *p* = 0.361), although the model remained significant overall (robust Wald *F* = 1.91, *p* = 0.032; *R*^2^ = 0.113; adjusted *R*^2^ = 0.047). Educational background remained a positive predictor (*β* = 0.288, *p* < 0.05), as did both experience categories (5–10 years: *β* = 0.601, *p* < 0.01; 10+ years: *β* = 0.610, *p* < 0.05). Perceptions of the public attenuated to marginal significance in this specification (*β* = 0.118, *p* < 0.10), and officers working in Hawalli were marginally more critical of *wasta* than those in the capital (*β* = 0.341, *p* < 0.10). Across the three models, adjusted *R*^2^ ranged from 0.026 to 0.047, indicating that the predictors explain a modest share of variance in officers’ attitudes toward *wasta*.

### 3.2 Interviews

#### 3.2.1 Circumstances

Discussion of circumstantial factors was prominent during interviews with officers. Discussion of illegitimate uses of *wasta* was associated with reasons related to Ease of Life (5 sources, 10 references) in attempts to secure more favorable outcomes (e.g., faster service times, bypassing prerequisites). Also included was a discussion of legitimate uses of *wasta*, ones that were heavily associated with Necessity (5 sources, 15 references), reflecting moral justifications where *wasta* is seen as a solution during hardship or injustice. Emerging sub-themes, such as Catalysts to *Wasta* (7 sources, 17 references) and Deterrents of *Wasta* (5 sources, 12 references), were prominent, illustrating the dualistic environments that spur or deter the prevalence of *wasta*.

Catalysts for *Wasta* were commonly associated with systemic and procedural inefficiencies that prompted officers to seek alternative pathways to achieve goals. Participants described *wasta* as a pragmatic tool for overcoming bureaucratic delays or securing timely access to services. On the other hand, Deterrents of *Wasta* emphasize the perceived risks associated with its misuse. Participants highlighted concerns about reputational damage, loss of professional credibility, and potential disciplinary action as key factors discouraging reliance on *wasta*. These deterrents were not merely formal or organizational but also rooted in personal values, indicating that officers are mindful of ethical boundaries, even within a culture where *wasta* is normalized.

This interplay between catalysts and deterrents underscores the context-dependent nature of *wasta*, suggesting that decisions to engage in the practice are shaped by a complex balance of cultural norms, moral considerations, and situational pressures. Such findings add nuance to our conceptual framework and support theoretical frameworks that conceptualize *wasta* as neither universally accepted nor inherently corrupt, but as a nuanced social mechanism shaped by competing moral, social, and practical imperatives.

#### 3.2.2 Capacities

The Capacity category had the fewest overall mentions, with just a few references in nodes like Rank or Status (4 sources, 7 references) and Social Connections (4 sources, 8 references). Although these factors indicate that hierarchical position and personal networks can support *wasta*, they appear more as enabling factors rather than primary causes. Other sub-nodes, such as Lack of Access and Unwillingness Toward *Wasta*, received minimal attention, reinforcing the idea that capacity-related factors are secondary to cultural and situational influences.

#### 3.2.3 Pressures

Pressures emerged as the most frequently cited category, especially concerning cultural norms. Societal Expectations (7 sources, 25 references) are the most referenced factor, highlighting strong normative obligations to engage in *wasta* as a display of loyalty and solidarity. Similarly, Boundaries (5 sources, 22 references) reflect an awareness of limits, showing that actors differentiate between acceptable and excessive forms of *wasta*. Internal and organizational pressures—though less prevalent—reflect a layered environment in which professional responsibilities intersect with cultural norms. These results support the view of *wasta* as a socially accepted practice rather than a personal deviation from established norms.

It should be noted that a previously unaccounted-for theme emerged during interviews. This included discussions of the perceived consequences of *wasta* in policing. ‘Police Work’ (7 sources, 18 references) is prominent, with respondents heavily noting the disruptive effects that *wasta* had on their roles as members of law enforcement. This was particularly tied to their responsibilities as higher-ranking officers, ones tasked with organizing and allocating members of the force effectively and efficiently. Table 5 presents the complete distribution of sources and references for each theme.

**Table 5:** Reference breakdown.

Section/node	Sources	References
Circumstances		
Catalysts to *Wasta*	7	17
Deterrents of *Wasta*	5	12
Illegitimate *Wasta*		
Ease of Life	5	10
Occupational	2	2
Legitimate *Wasta*		
Lack of Knowledge	1	1
Necessity	5	15
Wronged	5	6
Capacity		
Lack of Access	1	1
Rank or Status	4	7
Social Connections	4	8
Unwillingness Toward *Wasta*	1	1
Pressures		
Boundaries	5	22
External Pressure		
Organizational Pressures	3	6
Social Expectations	7	25
Internal Pressure	4	7
Doing What’s Right	2	3
Personal Ambitions	2	2
Consequences		
Police Work	7	18
Public Support	1	2

The following table exhibits qualitative findings from semi-structured interviews conducted with officers in Kuwait (*n* = 7). Sections are themes that were deductively included through this research’s predetermined framework for *wasta*. Nodes were inductively uncovered during the interview processing, forming sub-themes. The sources column identifies how many interviews to which sub-themes were brought up or discussed, while the references column includes the total instances where each sub-theme was discussed across all interviews conducted.

## 4 Discussion

This study aimed to examine the prevalence of *wasta* within Kuwait’s police force, using a mixed-methods approach to provide a robust investigation of attitudes toward its prevalence in Kuwaiti policing. Quantitatively, significant results were identified, showing that negative attitudes toward *wasta* correlate with officers’ educational background, years of experience, and public perceptions. Meanwhile, qualitative findings provided nuance behind findings while also identifying aspects previously unaccounted for in this research’s proposed framework. These findings offer valuable insights, marking the first study to primarily focus on *wasta* and its embeddedness in Kuwait’s police culture. The following sections will discuss these findings. Results found in both methodologies were first discussed; afterward, relevant findings from each will be elaborated on.

### 4.1 Duties to the public and *wasta*

The findings revealed that sampled officers with more positive views of the public were more likely to adopt a critical stance on *wasta* and its role in policing. This association should be interpreted carefully and in both directions: officers who view the public more favorably may be more likely to see *wasta* as incompatible with their duties. Conversely, officers with more permissive attitudes toward wasta might also hold fewer positive views of the public. It’s important to note that, since the survey measured attitudes rather than actual participation, we cannot conclude whether officers are actively involved in *wasta* from this relationship alone. Qualitative findings on ‘Boundaries’ support this, with most officers interviewed condemning *wasta* as a serious threat to justice, which is closely linked to law enforcement responsibilities. Officers 3 and 5, respectively:

Officer 3: “I set the bar when members of the public are actively harmed by its (wasta’s) use. I will actively fight it and do my utmost in helping individuals negatively affected by it.”

and

Officer 5: “The most important thing is to have a heart for the job and dedication to its core principles. The people who like wasta?” It’s those who are in the job for all the wrong reasons.”

Officers firmly committed to upholding justice would presumably resist integration into *wasta* culture, recognizing it as fundamentally unjust and at odds with their professional responsibility to serve and protect the public (Westmarland, 2005). It can also be argued that officers engaging in *wasta* are more likely to have negative encounters with members of the public, often resulting in allegations of misconduct for their participation in *wasta*, thereby damaging public perceptions of the police (Wolfe and Nix, 2016). These factors contribute to the deepening entrenchment of a distinct police culture sustained by such practices.

It should also be noted, however, that officers overwhelmingly agreed that there is a necessity to use *wasta*. This was particularly relevant in instances where individuals were forced to use it to navigate bureaucratic systems that posed hurdles to even the most basic services (e.g., driver’s licenses, birth certificates, authentication). This was extensively detailed by Officer 7, who added:

Officer 7: “Wasta is so ingrained that it even reaches the point that even if your transaction is done, there is still pressure—or they deliberately create pressure in the service departments—so that the person in charge of your transaction elongates it on purpose. Something that takes ten minutes becomes three or four days.”

This highlights the need to discuss the perceived status quo and its link to *wasta*. The conversation about how widespread wasta is reveals that *it is* viewed as a defined part of Kuwait’s policing system, one that cannot be simply fixed through self-correction. This is similar to clientelist systems observed in various countries, such as *Guanxi* in China, *Blat* in Russia, and *Jaan-Pechaan* in India^2^ (Berger et al., 2019).

### 4.2 Experience and *wasta*

Also exhibited in the results were the professional characteristics of officers and their potential impact on attitudes toward *wasta*. The findings revealed that officers’ experience strongly and positively correlated with the acknowledgment of *wasta* as detrimental to policing. This suggests that more experienced officers are more aware of the problems associated with *wasta* within Kuwait’s police force, which aligns with prior literature. These results were corroborated by qualitative findings, with a majority of officers indicating that they identified *wasta* as a detrimental aspect, particularly in administrative contexts. Officers 1 and 6 specifically elaborated on these areas, commenting:

Officer 1: “You realize its negatives mostly when dealing with administrative work. How are you supposed to organize everything when wasta keeps ruining organization and plans set in place? This includes administrative work, coordination and distribution of manpower.”

and

Officer 6: “Of course, when I work in a department in which half of the employees are transferred out through wasta, I’m forced to work extra shifts because of the shortage or inadequate work. These employees are the ones you can’t trust with finishing the job, something that makes my role as a leader very difficult.”

These findings have been supported by existing literature. Ingram et al. (2018) noted that senior officers were more likely to recognize the negative implications of rigid police cultural norms, such as the code of silence. Paoline and Gau (2018) further elaborated on these cultural norms using a monolithic model, finding officers with over 10 years of experience to be notably critical of the negative influence police culture exerts on police-public relations. Similarly, Charman (2017) observed that officers initially accepted police culture but gradually became disillusioned due to elements such as cynicism, group conformity, and critical views toward the public. In police stations, seniority often governs the distribution of responsibilities, with various shifts overseen by different senior officers. The culture of *wasta* compromises police effectiveness within these stations, adding occupational stress to senior officers (Brown et al., 2001). These senior officers are also subject to expectations to uphold *wasta*, particularly from less senior and less experienced officers who lack the practical experience necessary to fully experience its detrimental effects. This raises concerns about *wasta’s* role as a catalyst for divisions within Kuwait’s police institutions, creating a cycle in which tensions between senior officers and newly assigned recruits are reinforced by preconceived assumptions about *wasta* and the benefits less experienced officers may expect. This dynamic can significantly undermine group solidarity and negatively impact overall officer effectiveness (see Brough et al., 2016).

### 4.3 Officer backgrounds and attitudes toward *wasta*

Officers with college degrees demonstrated greater awareness of the detrimental effects of wasta within the police force. Degree-holding officers are often characterized as more mature and proactive in their policing approaches, and prior research has noted their resilience against traditional norms prevalent in police culture (Cox and Kirby, 2018). Additionally, scholarly literature highlights that degree holders often exhibit transformational leadership qualities, reflecting greater openness to community-oriented policing practices (Rydberg and Terrill, 2010). In Kuwait, prospective commanding officers entering the academy with only a high school diploma undertake four-year training programs comparable to European police training standards (e.g., Finland’s 3-year Police University program). They reside on the academy grounds for the majority of their training.

Conversely, candidates already holding a college degree are offered accelerated training programs intended to incentivize the recruitment of highly educated individuals into policing roles. These recruits, identified as specialized cadets, typically form smaller cohorts and experience relatively minimal police academy training and socialization. Beyond considerations of educational attainment and prior college socialization as foundations for superior policing, attention should also be given to their reduced exposure to academy socialization, which typically serves as an entry point into police cultural indoctrination (Doreian and Conti, 2017). This difference in academy exposure raises important implications regarding the influence of prolonged academy settings on officers’ integration into police culture. It is worth noting that the qualitative findings neither denied or confirmed these quantitative findings, despite the fact that three of the seven interviewed officers were college graduates.

### 4.4 Deterrents and catalysts to *wasta*

The qualitative portion of this research drew heavily on discussions of elements that both spur and combat the perpetuation of *wasta*. These discussions were mainly centered on the resilience of different agencies to *wasta*, or at least on elements that actively deter its perpetuation. Similarly, these discussions provided insights into specific sectors considered hotbeds of *wasta*, often targeted by individuals seeking to engage with its ecosystem. For instance, Officer 2—an individual working in investigations—commented on how use of *wasta* in departments was very risky and often avoided due to the sensitive nature of his work. He commented:

Officer 2: “In my line of work, it’s a very slippery slope. Most of our work involves official documents and testimony; hence, the capacity of any given person in our field to alter or provide wasta is extremely risky. The law is clear, and no one is going to put themselves in the line of fire just to appease someone.”

This contrasts with the opinions of sectors called ‘service sectors’, which are deemed to have the most prevalent *wasta* culture according to officers’ interviews. The vast number of bureaucratic processes found in these sectors serves as a goldmine to those looking to exploit them. Officer 6 elaborated:

Officer 6: “If it's a service sector, know well that there is a high percentage chance they like receiving calls for wasta. People will definitely come knocking on your door, regardless of whether you want to participate (in wasta) or not”

As for the discussion on the deterrents of *wasta*, there was a unified discussion of recent attempts at digitization and the Kuwait Police’s campaign to combat overly bureaucratic processes that previously required physical paperwork to be signed. Officer 4 commented on this:

Officer 4: “E-forms now facilitate all of these (previously bureaucratic) services, and they (perpetuators of wasta) are all struggling because it's all evolving. It means you don't need to wait for a particular individual to approve a form that meets all the necessary prerequisites. With the development of the police force, the precedent of wasta disappears.”

The literature on government processes and their association with ineffectiveness and inefficiency resulting from bureaucratic approaches has been widely studied across various fields. For instance, Castro and Lopes (2022) highlight how the use of information and communications, mainly through e-government, significantly combats corruption and bureaucratic inefficiency. They argue this comes through diminishing the discretionary power of officials, thereby reducing opportunities for corruption. Similar to Kuwait, Ramaswamy and Selian (2009) discuss legacy bureaucratic processes and their embeddedness in corrupt practices. They emphasize the need for carefully planned transitions and government processes to ensure that these practices are effectively combated through effective restructuring. Hence, initiatives in Kuwait and neighboring nations should focus on modernizing processes and minimizing discretionary instances, which serve as ripe environments for the perpetuation of *wasta*. This should be prioritized in so-called ‘service sectors’ where interactions with members of the public are abundant.

### 4.5 Social pressures

The most frequently discussed theme and sub-theme during interviews was the role social pressures play concerning *wasta*. All of the officers interviewed spent considerable time discussing the external pressures they faced as a result of *wasta* requests from members of the inner circle or individuals associated with them. Officer 4 discussed how the centralized nature of Kuwaiti society catalyzes these pressures:

Officer 4: “We are incomparable to any country outside of the Gulf community. We are a close-knit community with customs and traditions. We have a close-knit tribal group that remains close to this day. Everyone is easily reached in Kuwait by this connectivity.”

This close-knit nature was similarly discussed by Officer 1, who commented on the consequences of declining requests.

Officer 1: “You're going against the grain; you're going against society. In his eyes, you are a person who is of no use to your close ones because you are going against their potential benefit.”

Officer 7 even provided more detail to this phenomenon, arguing that even individuals outside one’s close circle can exert pressure on officers through their family members.

Officer 7: “It gets to the point where people start avoiding or cutting ties with my father and uncles if I reject offers (of wasta). What am I supposed to do in this case? I’m forced to do what I can to make sure they aren’t ostracized.”

These issues are heavily influenced by traditional elements discussed by Cunningham et al. (1994), with obligations to groups or sects serving as paramount for many individuals who consider themselves part of them. These issues heavily weigh on officers the more responsibility and power they wield, regardless on whether they subscribe to *wasta* culture or otherwise (AlSabah et al., 2025b). This establishes an ecosystem in which even more open-minded individuals seeking to break away from occupationally toxic norms, such as *wasta*, are forcibly pigeonholed into participating (Aldossari and Robertson, 2016). This calls into question the unfairness of social contracts associated to *wasta*, ones that can be unfair and taxing on individuals working in high-value areas when factoring in capacities toward providing desired services.

### 4.6 Updated framework of *wasta*

The qualitative findings improved the proposed framework by clarifying how circumstances, capacity, pressures, and response interrelate. Informed by these findings, the revised framework now includes catalysts, deterrents, occupational context, network proximity, and boundaries, which became more evident or emerged during the interviews.

In these situations, the difference between legitimate and illegitimate uses stays crucial. Yet, the results imply they might reinforce each other. Frequent illegitimate use can lead to delays, disadvantages, and an institutional culture in which people see wasta as essential to achieving lawful results. Bureaucratic inefficiency fuels this cycle, but digitization and formal protections can lessen reliance on informal measures.

The findings confirm the understanding of capacity. Direct capacity is linked to a person’s role, authority, and professional sector, with certain tasks providing more discretion. Indirect capacity works through social networks, encompassing colleagues, family, rank, status, and intermediaries. Thus, capacity indicates access to influence rather than a desire to leverage wasta.

Once access is granted, both internal and external pressures shape the potential provider’s actions. These pressures can include loyalty, reciprocity, personal motives, societal expectations, family obligations, organizational demands, and the social consequences of refusal. The provider’s ultimate decision is influenced by boundaries such as professional ethics, the potential for public harm, reputational and disciplinary risks, and institutional controls, such as digitization. The dynamic interaction between these pressures and boundaries ultimately decides whether the request is accepted or rejected.

**Figure 2:**
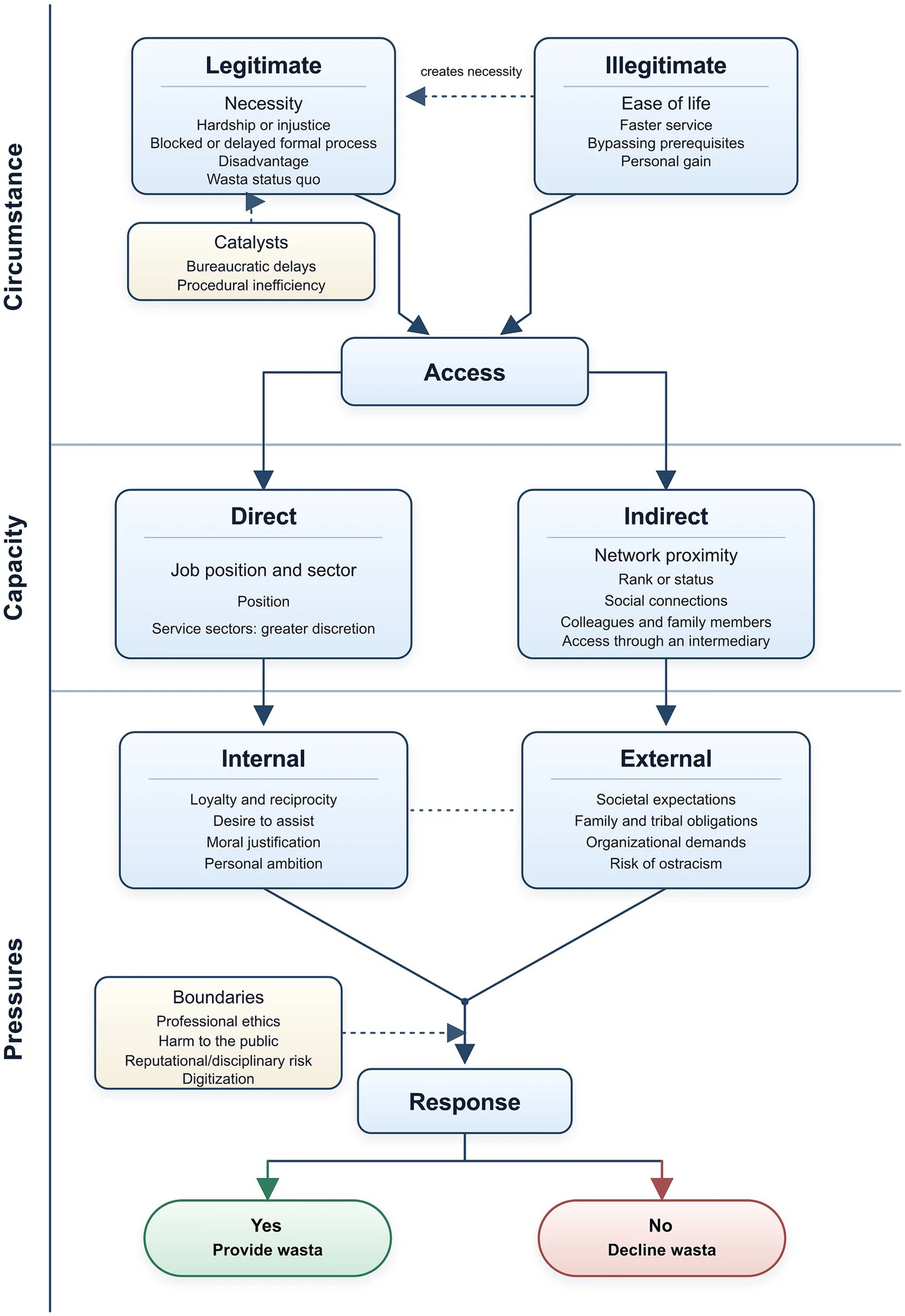
Updated wasta conceptual framework. The following figure presents an updated framework that adds nuance to pre-existing pillars identified through qualitative interviews.

The revised framework (see Figure 2) conceptualizes wasta as a conditional process driven by the interplay of institutional factors, access to capable actors, social obligations, and constraints on provider discretion. It also highlights several potential intervention points for reform, such as minimizing procedural inefficiencies, limiting unnecessary discretion, enhancing professional safeguards, and reducing dependence on personal connections.

### 4.7 Limitations

Although this study uses one of the largest samples to date on officers’ views of this topic, the sample remains limited in scope. A larger sample would yield more robust and precise findings. Additionally, participants in the quantitative portion of this research were exclusively from police stations, which, while the largest sector of Kuwait’s National Police, limits generalizability. Station environments differ from other divisions: they involve group-based work, frequent public interactions, and often negative encounters, such as dealing with suspects or detainees. These dynamics may heighten exposure to *wasta* and foster more critical attitudes toward the public.

Also worth noting is the internal makeup of the quantitative sample. It was uneven, with over half of respondents being high-ranking commanding officers and about two-thirds having over 10 years of service. This skew is due to combining a leadership-focused dataset with a general officer sample. The overrepresentation of senior personnel limits the ability to generalize these findings to the entire force and suggests that the links observed between rank, experience, and attitudes toward *wasta* might partly reflect the organizational hierarchy and officers’ roles within it, rather than personal attitudes alone. Therefore, any broad conclusions from these results should be tempered.

Moreover, the sampled officers were all male. This does not reflect the absence of female officers from Kuwait’s National Police; rather, it is due to women not yet being assigned to police stations within the Public Security Sector. This gap is significant: as a male-dominated institution, law enforcement often expects male members to engage in wasta, an informal institution spanning decades. This, however, might not be the case for female officers, given their inclusion in the police force—approximately 10 years ago—and their significantly lower numbers, which might exempt them from participation in such institutions. Additionally, access to these informal networks may be difficult for female members of law enforcement, a byproduct of the region’s male-dominated criminal justice systems (see AlSabah et al., 2026). Nevertheless, future studies should look to integrate female officers to better understand gender dynamics and their interplay with wasta.

Notable qualitative limitations were also present. First, using a non-probability sampling method raises concerns about bias, as participants may not fully represent the broader officer population. Although efforts were made to include officers from both administrative and field sectors, this limitation must still be addressed. It should also be noted that although saturation was achieved through interviews, the small sample size (*n* = 7) and voluntary participation limit the diversity of perspectives, potentially affecting findings.

It’s also important to note that the qualitative analysis was conducted by a single researcher. There was no second coder to review the transcripts, so intercoder reliability could not be assessed, and no formal peer debriefing or member checking was performed. As a result, the subthemes and reference frequencies listed in Table 5 are based solely on one analyst’s interpretive judgments and might not be exactly reproducible by others. Since the parent themes were identified deductively, this method has some risk of confirmation bias, although the discovery of an unexpected theme (Consequences) indicates that the coding process remained data-driven. Future research should include team-based coding, formal intercoder reliability assessments, and participant validation of the themes.

Lastly, due to the sensitive nature of *wasta*, social desirability bias could have occurred, causing participants to underreport personal involvement or exaggerate their opposition to the practice. This risk is more pronounced in a policing setting, where revealing involvement might lead to professional consequences. To address this, anonymity guarantees, private phone interviews, and reminders of the right to withdraw were employed to reduce—but not fully eliminate—bias. Likewise, using convenience sampling for interviews could have attracted officers with more extreme opinions on *wasta*, whether positive or negative, compared to the typical member of the force.

### 4.8 Conclusion

These findings address gaps in understanding wasta in Middle Eastern criminal justice, with implications for reform. Structural reforms like digitizing procedures and increasing transparency can limit discretionary authority and reduce delays. Cultural changes, including ethics training and community policing, promote meritocracy and honesty. Combating wasta needs policy and cultural shifts to transform organizations and ease social pressures. While tested in Kuwait, the framework’s concepts apply broadly, reflecting institutional friction, network proximity, and normative pressures. Future research can empirically evaluate its effectiveness across similar informal practices like guanxi or blat.

Furthermore, this study lays the groundwork for future research within the broader criminal justice system in the Middle East, particularly investigations into cultural dynamics that influence the effectiveness of justice enforcement in local communities. Through its reinforced conceptual framework, future studies are provided with proven themes to base their research on, offering an informed foundation on wasta use in criminal justice—one applicable to other fields as well. Ultimately, this research emphasizes the critical importance of addressing culturally embedded practices, such as *wasta*, to enhance institutional integrity, strengthen police-community relationships, and improve overall policing effectiveness, both in Kuwait and in similar contexts throughout the Middle East.

## Data Availability

The raw data supporting the conclusions of this article will be made available by the authors, without undue reservation.

## Footnotes

1 It is important to note that these jurisdictions—spanning from the UK’s consent-based, constable-driven policing tradition to Russia’s centralized federal system—are fundamentally different in their legitimacy principles and their relationships between the state and society. They are mentioned here not as directly comparable systems but as separate examples of a wider legitimacy and integrity issue similar to *wasta*.

2 These are parallel expressions of informal exchange, not exact equivalents.
