# Supplementary material for: Kinship, favors, and officer integrity: exploring wasta in Kuwait’s National Police Force
Source: Front Psychol. 2026 Aug 17;17:1849882. doi: 10.3389/fpsyg.2026.1849882 (PMC13526545; doi:10.3389/fpsyg.2026.1849882)
Supplement: Supplementary file 1 [file Table_1.DOCX]

Supplementary Material

Supplementary List 1:

1. **Demographic Background**
   - Participants were first asked basic demographic questions, including age, years of experience, rank, current work location, area of residence, education status, and marital status.
2. **Circumstances**
   - Under what circumstances is wasta most commonly used within the police force?
   - Can you share examples where wasta influenced decisions or resource allocation?
   - What organizational, social, or environmental factors contribute to the need for its use?
3. **Capacity**
   - How are officers usually contacted for wasta?
   - What personal traits or social connections increase a person’s ability to use wasta?
   - What barriers prevent others from engaging in or benefiting from it?
4. **Pressures**
   - What internal (e.g., ambition, workplace norms) and external (e.g., tribal or familial obligation) pressures lead individuals to participate in wasta?
   - Can you provide examples of how these pressures manifest in practice?
   - How do these pressures affect individual career progression or overall police organizational performance
